# Tribological Behavior of PEEK/PTFE Composites Reinforced with Carbon Fibers and Graphite

**DOI:** 10.3390/ma15207078

**Published:** 2022-10-12

**Authors:** Yingji Li, Yi Chen, Yongxin Guo, Da Bian, Yongwu Zhao

**Affiliations:** 1 School of Mechanical Technology, Wuxi Institute of Technology, Wuxi 214121, China; 2 Suzhou Nilfisk R&D Co., Ltd., Suzhou 215000, China; 3 College of Automation, Wuxi University, Wuxi 214105, China; 4 College of Mechanical Engineering, Jiangnan University, Wuxi 214122, China

**Keywords:** PEEK/PTFE composites, carbon fibers, friction coefficient, wear

## Abstract

In this study, poly (ether ether ketone) (PEEK)/polytetrafluoroethylene (PTFE) composites reinforced with carbon fibers (CFs) and graphite (Gr) were fabricated by compressive molding technology. The friction and wear properties of the PEEK/PTFE composites sliding against Si_3_N_4_ balls were investigated using ball-on-disk configuration under dry sliding conditions, and the morphologies of the worn surfaces were also observed with a scanning electron microscope (SEM) and a three-dimensional morphometer. The results indicated that the introduction of CFs significantly improved the tribological properties of the composites, but the friction coefficient of the PEEK/PTFE/CFs composites were higher than the pure PEEK/PTFE composites. However, it was found that a combinative addition of CFs and Gr creates an obvious synergetic effect of improving the friction-reducing and anti-wear abilities of the composites. The mechanisms of the improved tribological properties of the PEEK/PTFE/CFs/Gr composites were discussed based on the analysis of the worn surfaces and tribofilms.

## 1. Introduction

Poly (ether ether ketone) (PEEK) has been widely used in various fields, such as the automotive and aerospace industries, due to its outstanding mechanical properties, high thermal stability, high chemical resistance, and high wear resistance [1,2,3]. However, pure PEEK always exhibits a high friction coefficient and wear rate in dry sliding, which restricts its tribological applications [4,5,6].

Many researchers have found that the addition of appropriate fillers is an effective method to reduce the friction coefficient and wear rate of PEEK, such as solid lubricants and nanoparticles [7,8,9]. PTFE is considered as one of the best fillers to improve wear resistance of PEEK-based composites. The main factor affecting the tribological performance is the formation of a transfer film on the wear track, which can reduce the friction coefficient by avoiding direct contact between the friction pairs [10,11,12]. However, this approach comes at the cost of decreased mechanical strength and an increased viscosity of the melted composite, which might limit their use under severe loading conditions. It is known that the excellent self-lubricating and tribofilm-forming properties of the PTFE composite could be modified by the addition of fillers, such as carbon fibers, graphite, and ZrO_2_ [13,14]. Xie et al. [15] found that potassium titanate whiskers (PTW) were an effective reinforcement for PEEK/PTFE composites, and the microhardness of the composites was enhanced. Furthermore, the addition of PTW could improve the mechanical and tribological properties of the PEEK/PTFE composites. It was shown that the addition of PTW could greatly improve the wear resistance and reduce the friction coefficient of the composites. Lin et al. [16] studied the effect of various fillers (i.e., nano-sized, micron-sized particles, and short cut carbon fibers) on the PEEK-PTFE-steel hybrid wear system, without damaging its mechanical and processability properties. However, the detailed and specific influence of different types of filler in PEEK/PTFE composites remains unclear.

The purpose of this study is to develop a low friction and wear rate of PEEK/PTFE composites by the addition of carbon fibers (CFs) and graphite (Gr) by compressive molding technology. Tribological tests were performed to study the synergistic effect of CFs and Gr on the mechanical and tribological properties of PEEK/PTFE composites. Furthermore, the wear mechanisms of the PEEK/PTFE composites were analyzed.

## 2. Experimental

### 2.1. Materials

PTFE emulsion containing 60 wt.% of the PTFE resin (JF-4TN, nominal diameter 30 ± 5 μm) was purchased from Zhejiang Juhua Co., Ltd. (Quzhou, China). PEEK powder (085G, nominal diameter 20 μm) was purchased from Changchun Jida Special Plastic Engineering Research Co., Ltd. (Changchun, China). CFs (ZL-CF200, nominal diameter 7 μm) were produced by Zhongli New Materials Co., Ltd. (Cangzhou, China). Gr (XLJ-100, nominal diameter 15 μm) was purchased from Shanghai Xili Carbon Co., Ltd. (Shanghai, China).

### 2.2. Preparation of PEEK/PTFE Composites

PEEK/PTFE composites with dog-bone plates were produced using an injection molding machine (VI-55DRES, Guangzhou Zhongtai Precision Machinery Co., Ltd., Guangzhou, China). The thickness of the plates was 4 mm. The detailed compositions of all composites are listed in Table 1. The mass ratio of PEEK and PTFE was maintained as 19:1. The barrel temperature was set as 370 °C, and the mold temperature was kept at 175 °C.

### 2.3. Characterization

The mechanical properties of samples were tested using a universal testing machine. At least three measurements were performed to ensure the repeatability of the results. Tribological tests were performed under dry sliding conditions using a ball-on-disk configuration in the linear reciprocating mode (MFT-5000, Rtec, San Jose, CA, USA). Si_3_N_4_ balls with a diameter of 9 mm were used as the counterpart. The test parameters were set under the following conditions: applied load of 50 N; a frequency of 2 HZ; and a test duration of 20 min under air condition. The morphologies of the worn surfaces were observed with a scanning electron microscope (SEM, ZEISS EVO18, Oberkochen, Germany) and optical profilometry (MFP-D, Rtec, San Jose, CA, USA).

## 3. Results and Discussion

**Figure 1:**
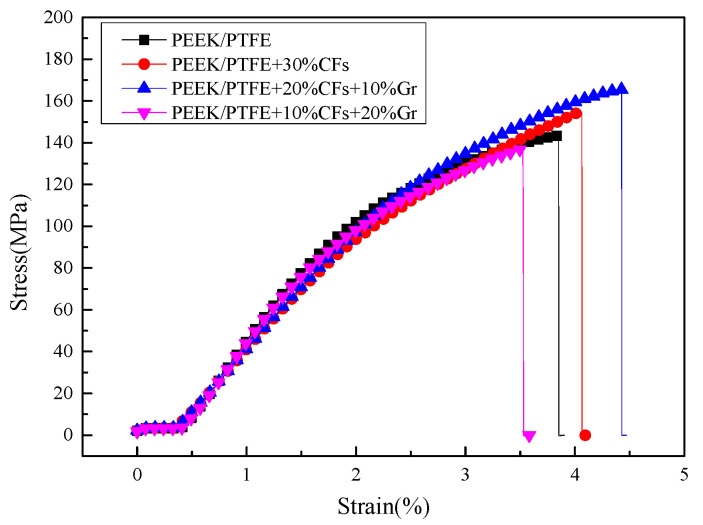
The stress-strain curves of four different PEEK/PTFE composites.

The mechanical properties of the composites were evaluated by tensile testing. The stress-strain curves of the composites are shown in Figure 1. Here, it can be seen that the tensile stress increases from 137 MPa to 155 MPa, by the addition of 30% CFs in PEEK/PTFE composites. Furthermore, it is higher than PEEK/PTFE composites with 10% CFs and 20% Gr, due to defects caused by the high content of Gr. However, the PEEK/PTFE composites exhibit the largest tensile stress when 20% CFs and 10% Gr are added into the composites. This means that CFs and Gr have a synergistic effect on the PEEK/PTFE composites.

**Figure 2:**
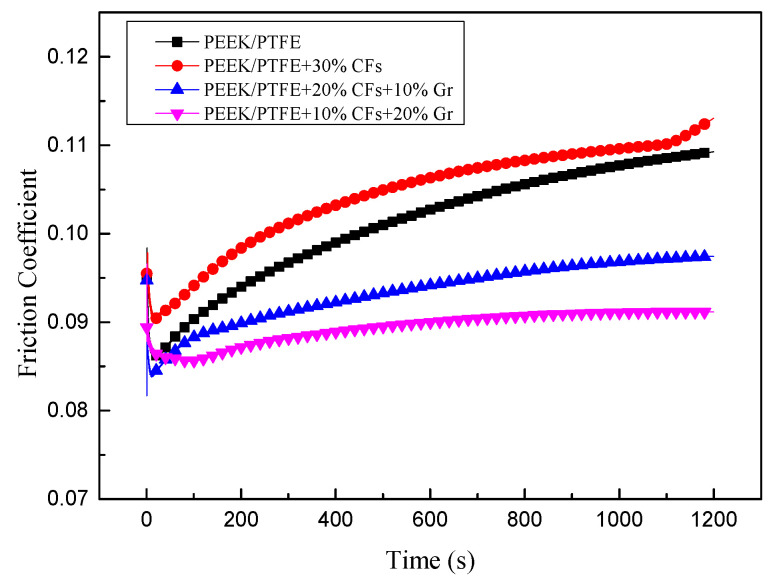
Friction coefficients of PEEK/PTFE composites reinforced with different contents of CFs and Gr.

Figure 2 shows the friction coefficients of the PEEK/PTFE composites with different contents of CFs and Gr. The result shows that the PEEK/PTFE composites show a similar friction coefficient at the beginning of the test. The difference in friction coefficient becomes obvious as time increases. It can be seen that the PEEK/PTFE composites with 30% CFs achieves a higher friction coefficient than the pure PEEK/PTFE composites, which is attributed to the additional abrasive wear caused by the CFs debris remaining in the wear track [17]. Furthermore, the friction coefficient decreases with the synergistic effect of CFs and Gr. The PEEK/PTFE composites with 10% CFs and 20% Gr achieves the lowest value among the tested samples.

**Figure 3:**
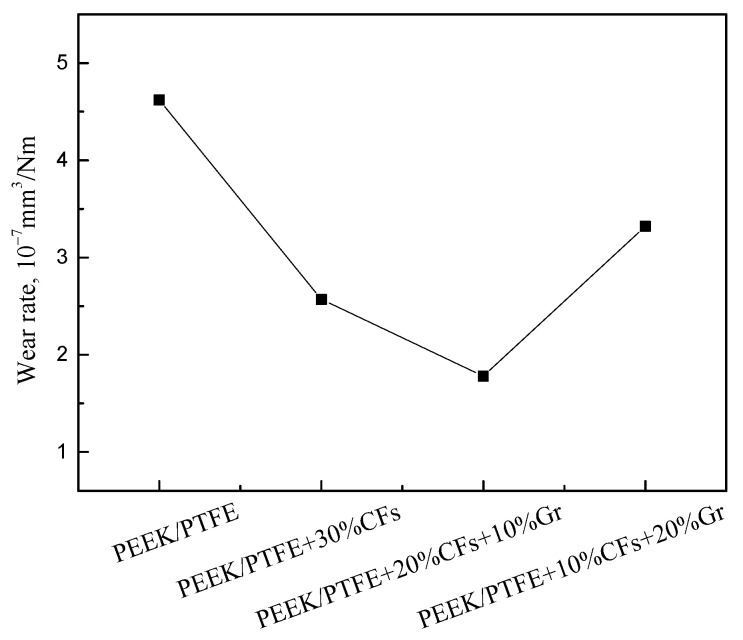
Wear rates of PEEK/PTFE composites reinforced with different contents of CFs and Gr.

The wear rate of the PEEK/PTFE composites was calculated and is shown in Figure 3. The wear rate of the pure PEEK/PTFE composites is about 4.62 × 10^−7^ mm^3^/Nm. In the meantime, the wear rates of the PEEK/PTFE composites significantly decrease with the addition of CFs and Gr. This clearly shows that the PEEK/PTFE composites with the addition of 20% CFs and 10% Gr exhibit the lowest wear rate compared to other samples. However, it should also be noted that the wear rate of the PEEK/PTFE composites with an addition of 10% CFs and 20% Gr is the second largest among the tested samples. This indicates that a high content of Gr will reduce the density of the PEEK/PTFE composites, and the Gr debris will retain in the wear track to decrease the friction coefficient as a solid lubricant. 

**Figure 4:**
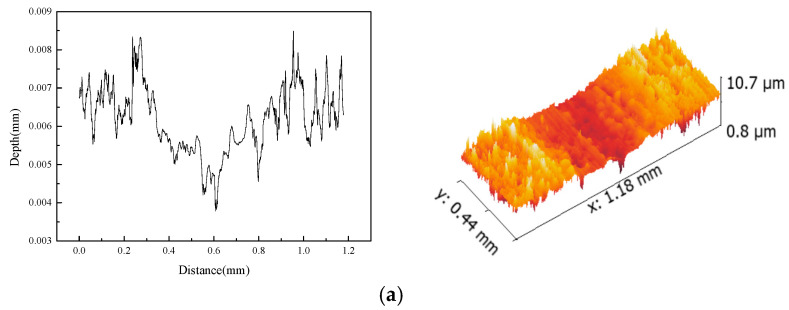

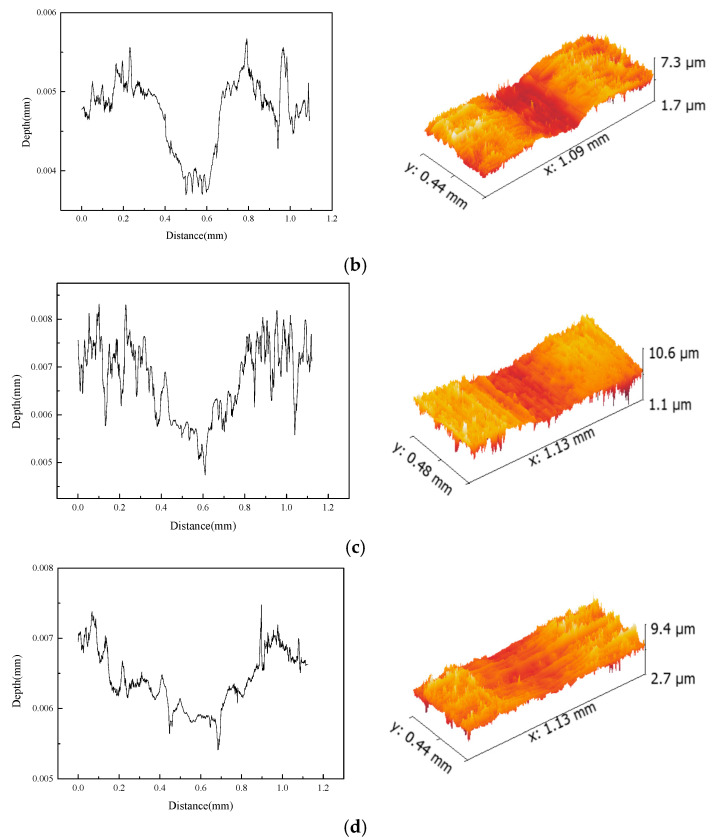
3-D morphologies and section profiles of the wear tracks of PEEK/PTFE composites: (**a**) PEEK/PTFE; (**b**) PEEK/PTFE + 30% CFs; (**c**) PEEK/PTFE + 20% CFs + 10% Gr; (**d**) PEEK/PTFE + 10% CFs + 20% Gr.

Figure 4 shows 3-D topographies of the worn surfaces of PEEK/PTFE composites reinforced with different contents of CFs and Gr. It is found that the pure PEEK/PTFE composites show some asperities, which are caused by the adhesive wear of the friction pair on the worn surface. However, the wear mechanisms show obvious differences with the addition of CFs. Figure 4b shows severe micro-cutting on the worn surface, which is the characteristic of abrasive wear. However, the width of the wear track is smaller than the pure PEEK/PTFE composites and leads to a smaller wear rate. This phenomenon is alleviated by the addition of Gr (Figure 4c), which shows less severe micro-cutting compared to that without Gr. Because of this, the wear rate of the PEEK/PTFE composites decreases. In addition, the width of the wear track (Figure 4d) becomes larger with the addition of a high content of Gr, which leads to a high wear rate.

**Figure 5:**
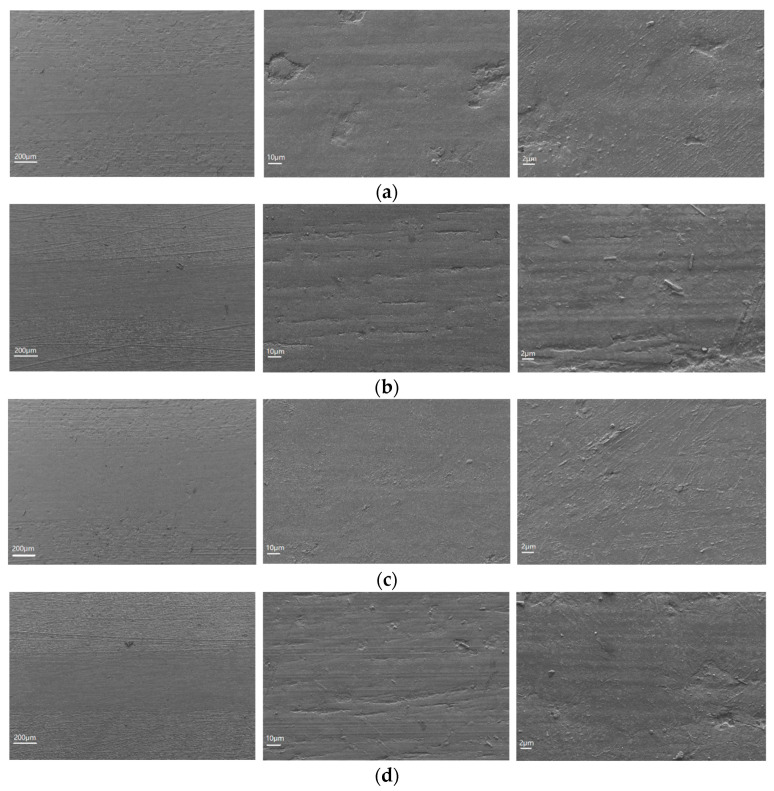
SEM micrographs of worn surfaces PEEK/PTFE composites: (**a**) PEEK/PTFE; (**b**) PEEK/PTFE + 30% CFs; (**c**) PEEK/PTFE + 20% CFs + 10% Gr; (**d**) PEEK/PTFE + 10% CFs + 20% Gr.

**Figure 6:**
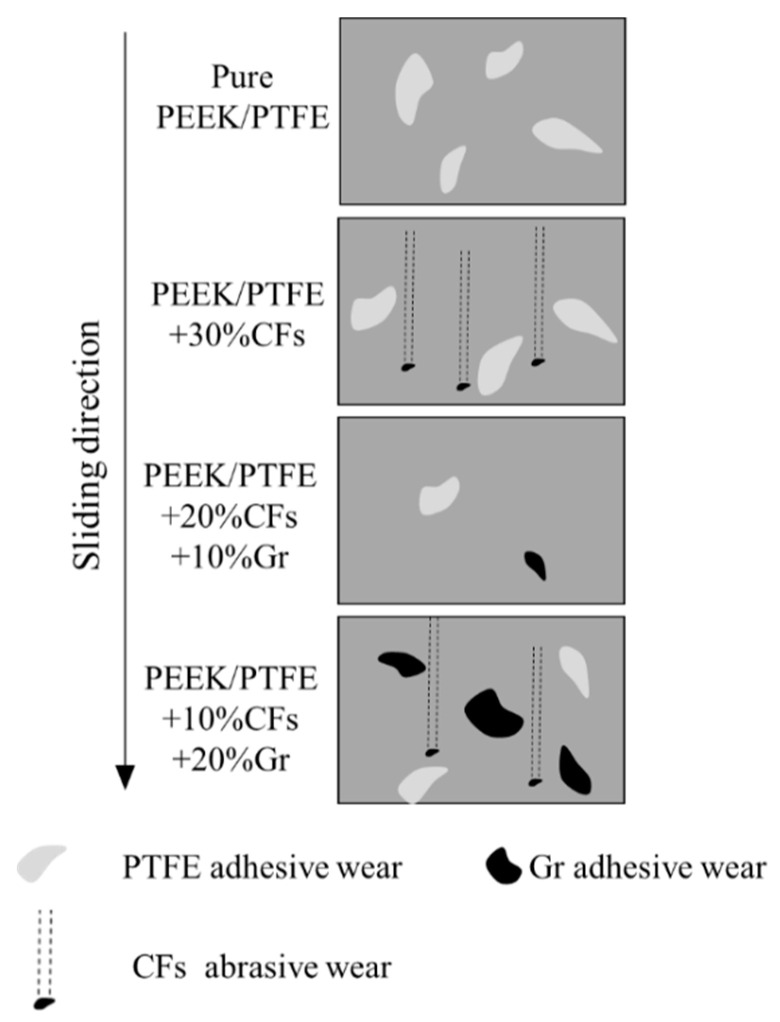
Schematic illustration of the wear mechanisms of PEEK/PTFE composites with different contents of CFs and Gr.

In order to study the influence of CFs and Gr on the wear mechanisms of the PEEK/PTFE composites, the worn surface morphology of the PEEK/PTFE composites reinforced with different contents of CFs and Gr were investigated with SEM, shown in Figure 5. The worn surface of the pure PEEK/PTFE composites was rough and characterized by severe scuffing (Figure 5a), which indicated that the dominant mechanism may be severe adhesion mechanism [18]. The worn surface of PEEK/PTFE composites with an addition of 30% CFs show a severe abrasion (Figure 5b). Furthermore, a large amount of CFs debris was observed on the worn surface, which was in line with the dramatic drop in wear resistance. The dominant wear mechanism for PEEK/PTFE composites with an addition of 30% CFs may be severe abrasive wear [17,19]. The worn surface of PEEK/PTFE composites with an addition of 20% CFs and 10% Gr was comparatively smoother and there was no obvious scuffing and spalling phenomena (Figure 5c). With the addition of Gr, a uniform and coherent transfer film was formed on the worn surface, and subsequently, a lower friction coefficient and wear rate was reached. However, the worn surface of PEEK/PTFE composites with an addition of 10% CFs and 20% Gr indicated slight adhesive wear and abrasive wear (Figure 5d). This was because the bonding strength of the PEEK/PTFE composites decreased with the addition of too much Gr. Therefore, CFs and Gr were released onto the wear track under contact pressure, which thus resulted in worse tribological properties. Based on the integrated analysis presented above, a schematic illustration of the wear mechanism of PEEK/PTFE composites with different contents of CFs and Gr is shown in Figure 6.

## 4. Conclusions

In this study, PEEK/PTFE composites reinforced with CFs and Gr were fabricated by compressive molding technology. The tribological properties of the PEEK/PTFE composites were evaluated and relative mechanisms were analyzed. Some conclusions were drawn as follows:

1. The PEEK/PTFE composites reinforced with 20% CFs and 10% Gr showed the highest tensile breaking force among all the samples. In addition, the PEEK/PTFE composites reinforced with 10% CFs and 20% Gr showed a lower value than the pure PEEK/PTFE composites.
2. The friction coefficient of the PEEK/PTFE composites increased with the single addition of CFs. Furthermore, the lowest friction coefficient was obtained by an addition of 10% CFs and 20% Gr, and the value was about 0.091.
3. The synergistic effect of CFs and Gr could greatly improve the wear resistance of the PEEK/PTFE composites. However, the high content of Gr led to a slight increase in the wear rate.
4. For pure PEEK/PTFE composites, the dominant wear mechanism may be severe adhesion mechanism. With the addition of CFs, the dominant wear mechanism transformed into an abrasive mechanism. The addition of CFs and Gr can improve the wear resistance of the PEEK/PTFE composites, so it can form a uniform and stable transfer film.

With the addition of CFs and Gr, the complete lubricating film could reduce the friction coefficient and wear rate of the PEEK/PTFE composites.

Overall, the addition of CFs and Gr were efficient in reinforcing PEEK/PTFE composites. These improved features of PEEK/PTFE composites are promising for advanced engineering applications.

## Figures and Tables

**Table 1:** The composition of the composites (wt.%).

Sample	PEEK/PTFE	CFs	Gr
PEEK/PTFE	100	0	0
PEEK/PTFE + 30% CFs	70	30	0
PEEK/PTFE + 20% CFs + 10% Gr	70	20	10
PEEK/PTFE + 10% CFs + 20% Gr	70	10	20

## Data Availability

Data is contained within the article.
